# Behavioral and Psychological Symptoms of Dementia as a Clinical Indicator of Disease Severity and Memory Decline in African Populations: Data from the READD–ADSP Project

**DOI:** 10.1002/alz70857_106579

**Published:** 2025-12-26

**Authors:** Olufisayo Oluyinka Elugbadebo, Gabriel O Ogunde, Joshua O Akinyemi, Temitope Hannah Farombi, Brian W Kunkle, Mayowa Ogunronbi, Larry D Adams, John‐Paul Umuojine, Adefolakemi Ogundele, Yared Z Zewde, Godwin Osaigbovo, Victoria Mutiso, Paul Olowoyo, Anyamele Ibuchim, Judith Boshe, Kamada Lwere, David Ndetei, Albert Akpalu, Michelle Nichols, Damas Andrea Mlaki, Fred Stephen Sarfo, Paul Nwani, Alfred K Njamnshi, Albertino Damasceno, Kolawole Wahab, Patrice G Whitehead, Reginald Obiako, Inna Logvinsky, Njideka U Okubadejo, Jovita D. Inciute, Stella‐Maria Paddick, Anthony J Griswold, Jacob L. McCauley, Raj N Kalaria, Jeffery M Vance, Oyedunni Arulogun, Olusegun Baiyewu, Goldie S Byrd, Adesola Ogunniyi, Margaret Pericak‐Vance, Rufus O Akinyemi, African Dementia Consortium

**Affiliations:** ^1^ University College Hospital, Ibadan, Oyo, Nigeria; ^2^ College of Medicine, University of Ibadan, Ibadan, Oyo, Nigeria; ^3^ Institute for Advanced Medical Research and Training, College of Medicine, University of Ibadan, Ibadan, Oyo, Nigeria; ^4^ Neuroscience And Ageing Research Unit, Institute for Advanced Medical Research and Training, College of Medicine, University of Ibadan, Ibadan, Nigeria; ^5^ University College Hospital, Ibadan, Nigeria; ^6^ John P. Hussman Institute for Human Genomics, University of Miami Miller School of Medicine, Miami, FL, USA; ^7^ University of Miami, Miami, FL, USA; ^8^ Komfo Anokye Teaching Hospital, Kumasi, Ghana; ^9^ Psychogeriatric Unit, Neuropsychiatric Hospital, Aro, Abeokuta, Nigeria, Abeokuta, Abeokuta, Nigeria; ^10^ College of Health Sciences, Addis Ababa University, Addis Ababa, Ethiopia; ^11^ Global Brain Health Institute, San Francisco, CA, USA; ^12^ Jos University Teaching Hospital, Jos, Plateau, Nigeria; ^13^ Africa Mental Health Research and Training Foundation, Nairobi, Kenya; ^14^ Ekiti State University Teaching Hospital, Ekiti, Ekiti, Nigeria; ^15^ Department of Medicine, University of Port Harcourt, Port Harcourt, Plateau State, Nigeria; ^16^ Kilimanjaro Christian Medical University College, Moshi, Tanzania, United Republic of; ^17^ Makerere University, Kampala, Uganda, Uganda; ^18^ University of Nairobi, Nairobi, Kenya; ^19^ University of Ghana Medical School, Accra, Ghana; ^20^ University of South Carolina, Charleston, SC, USA; ^21^ Mirembe National Mental Health Hospital, Dodoma, Dodoma, Tanzania, United Republic of; ^22^ Kwame Nkrumah University of Science and Technology, Kumasi, Ghana, Kumasi, Ghana; ^23^ Nnamdi Azikiwe University Teaching Hospital, Anambra State, Nigeria, Nnewi, Anambra, Nigeria; ^24^ Brain Research Africa Initiative, Yaounde, Centre, Cameroon, Yaounde, Centre, Cameroon, Cameroon; ^25^ Faculty of Medicine, Eduardo Mondlane University, Maputo, Mozambique; ^26^ Department of Medicine, University of Ilorin, Ilorin, Nigeria; ^27^ University of Miami Miller School of Medicine, Miami, FL, USA; ^28^ Neurology Unit, Department of Medicine, Ahmadu Bello University/Ahmadu Bello University Teaching Hospital, Zaria, Nigeria; ^29^ University of Miami, Miller School of Medicine, Miami, FL, USA; ^30^ College of Medicine, University of Lagos, Lagos, Nigeria; ^31^ Translational and Clinical Medicine, Newcastle University, Newcastle upon Tyne, United Kingdom; ^32^ Newcastle University, Newcastle, United Kingdom; ^33^ University of Ibadan, Ibadan, Oyo, Nigeria; ^34^ College of Medicine, University of Ibadan, Ibadan, Nigeria; ^35^ Wake Forest University School of Medicine, Winston‐Salem, NC, USA; ^36^ John P. Hussman Institute for Human Genomics, University of Miami, Miami, FL, USA

## Abstract

**Background:**

BPSD are common in persons living with dementia and are often associated with disease severity and caregivers’ distress. In Low‐ and Middle‐Income Countries, they are often the symptom complex that compel family to seek medical help. However, the association of BPSD with dementia severity and specific cognitive domains is underexplored in African populations. This study aims to evaluate BPSD among indigenous Africans and the association with specific cognitive domains.

**Method:**

This cross‐sectional study analyzes data collected from nine African countries (Nigeria, Benin, Ghana, Cameroon, Ethiopia, Kenya, Uganda, Tanzania, and Mozambique) participating in the Recruitment and Retention for Alzheimer's Disease Diversity Cohorts in the Alzheimer's Disease Sequencing Project (READD‐ADSP) project, from June 2023 to December 2024. Data on neurocognitive factors were collected using the Uniform Data Set (UDS), Neuropsychiatric Inventory Questionnaire (NPI‐Q) for BPSD, and the Clinical Dementia Rating (CDR) scale for dementia severity. Data was managed on REDCap and analysis was performed using STATA (v16). Descriptive statistics were employed for symptom prevalence, and Spearman's rank correlation was used to analyze the relationship between NPI‐Q and CDR scores. Association between cognitive domains and behavioral symptoms was examined using hierarchical linear model adjusting for covariates such as age, gender, education, and illness duration.

**Result:**

A total of 641 Africans with dementia (61% female, mean age 76.7±10.1 years) were included. The mean (±SD) NPI and CDR scores were 4.91 (±6.25) and 1.84 (±1.25), respectively. The most prevalent BPSD symptoms were apathy (32%), agitation/aggression (28.7%), and depression (28.1%). Spearman's correlation revealed a weak but significant positive association between NPI‐Q severity scores and CDR scores (rho = 0.264, *p* < 0.001). Behavioral symptoms were significantly associated with dementia severity (β = 1.59, CI: 1.15–2.03) as well as specific cognitive domains, particularly memory (digit span backward: β = ‐0.38, CI: ‐0.64 to ‐0.13). However, the duration of illness was not a significant predictor of behavioral symptoms in this cohort.

**Conclusion:**

The findings suggest that BPSD are both an indicator and consequence of dementia severity, particularly in memory function. This study posits the need for targeted management of BPSD and improvement in overall patient care in LMICs

